# Environmental Impacts of Cultivated (Lab-Based) Blue Foods

**DOI:** 10.1021/acs.est.6c02274

**Published:** 2026-05-26

**Authors:** Shira Shabtai, Tamar Makov, Alon Shepon, Patrik J. G. Henriksson

**Affiliations:** † Management Department, 26732 Ben-Gurion University of the Negev, Post Office Box 653, Beer Sheva 8410501, Israel; ‡ School of Sustainability and Climate Change, Ben-Gurion University of the Negev, Beer Sheva 8410501, Israel; § The Steinhardt Museum of Natural History, Tel-Aviv University, Post Office Box 39040, Ramat Aviv, Tel Aviv 6139001, Israel; ∥ The Department of Public Policy, The Faculty of Social Sciences, Tel Aviv University, Tel Aviv 6997801, Israel; ⊥ Institute of Environmental Sciences (CML), Leiden University, 2333 CC Leiden, Netherlands; # Stockholm Resilience Centre, 7675 Stockholm University, 114 19 Stockholm, Sweden

**Keywords:** cultivated meat, life cycle assessment, seafood, global warming, cell culture, aquatic foods, fish, eel

## Abstract

Lab-based meat or cultivated meat (CM) is produced by growing edible animal cells using cellular agriculture techniques and eliminates the need to farm or capture live animals. Given the depleted state of global fisheries and adverse environmental impacts associated with aquaculture, CM systems may be particularly suited to help meet the rising demand for blue foods, especially high-value and luxury species, sustainably. Despite growing research into CM products and their potential to lower the environmental impacts associated with animal-based foods, the environmental performance of CM blue foods remains largely unknown. Here, we use life cycle assessment (LCA) to quantify the cradle-to-gate environmental impacts associated with three CM blue food products. We find that CM blue foods have a cumulative energy demand of 40–45 MJ kg^–1^, emit 2.1–2.3 kg of CO_2_ equiv kg^–1^ (global warming), require 1.1–1.6 m^2^ a kg^–1^ (land use), consume 97–152 L kg^–1^ (water use), and contribute 0.9–1.3 g of P equiv kg^–1^ and 0.9–1.1 g of N equiv kg^–1^ (freshwater and marine eutrophication). These impacts are generally lower than those reported for cultivated farm animals and for conventionally produced analogous blue foods. However, results reveal potential for environmental trade-offs (e.g., higher water use) and underscore the need for multi-indicator environmental evaluation of novel foods.

## Graphical abstract

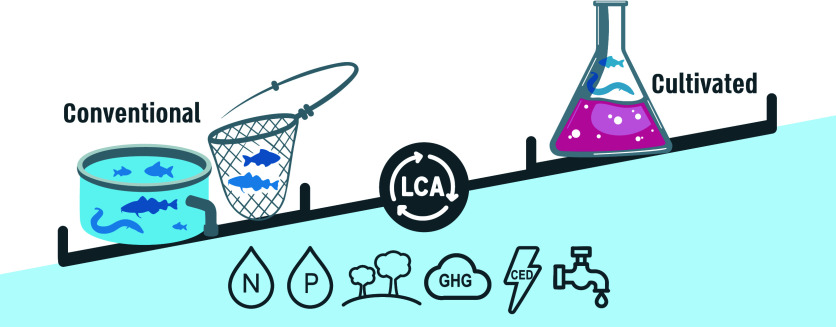

## 1 Introduction

Animal-based foods are associated with disproportionately large environmental impacts.^1^ Research suggests that globally, animal products are associated with over 80% of agricultural land use, 40% of agricultural water use,^2^ and 20% of global anthropogenic greenhouse gas (GHG) emissions.^3^ In addition, agriculture is the single largest contributor to biodiversity loss (via land use and land use change), with fishing the major driver of marine biodiversity loss.^4^ Predictions suggest that global demand for animal-based foods will increase by 63% over the next 20 years,^5^ highlighting the urgent need to find more sustainable ways to meet growing demand.

Cultivated meat (CM hereafter), also known as lab-based meat, cellular, cell-based, in vitro, or cultured meat, is a novel food production system often put forward as a more sustainable alternative to conventional production of animal-based foods.^6–10^ CM systems can produce various types of animal cells (e.g., livestock, poultry, or aquatic foods) in controlled environments, bypassing the need to farm or capture live animals.^11–13^ Though many technical challenges and open questions regarding issues such as economic feasibility, ethics, regulation, and consumer acceptance remain,^14–16^ proponents of CM often highlight the potential environmental benefits that could be achieved if CM replaces conventional animal-based foods.^6,10,17^ For example, Sinke et al.^10^ used life cycle assessment (LCA) to estimate that CM could result in 90% lower GHG emissions compared to conventional beef production. To date, however, no LCA study has estimated the environmental impacts associated with CM blue foods.

Blue foods encompass 3000 to 4000 aquatic species, including finfish, crustaceans, mollusks, and seaweeds, which are conventionally produced through capture fisheries (i.e., wild catch) or aquaculture.^18–20^ Global per capita consumption of blue foods is estimated at approximately 21 kg annually, providing an important source for many essential nutrients.^20^

Like terrestrial animal-based foods, conventional production of blue foods is associated with a wide range of environmental burdens.^21^ Capture fisheries are greatly dependent on fossil fuels^22^ and contribute to habitat degradation and biodiversity loss.^23–25^ Overexploitation of many fish stocks places them at risk of collapse,^26,27^ while an estimated 40% of global marine catches are bycatch, meaning nontarget species caught alongside intended species.^28^ The impacts of climate change further exacerbate these challenges, with predictions suggesting a decline in the quantity, size, and nutrient content of blue foods.^29^

Over the past few decades, stagnation in capture fisheries landings has shifted the supply of blue foods toward aquaculture, which today accounts for over half of global production.^20,31^ Conventional aquaculture practices have, in turn, been linked to numerous environmental concerns, including pollution, habitat degradation, and spread of invasive species, diseases, and parasites.^21^ Moreover, most aquaculture still exerts pressure on wild fisheries through its reliance on wild-caught fish for feed and juveniles (fishmeal and fish oil).^31,32^ Thus, as global demand for blue foods is projected to double by 2050,^33^ production expansion and subsequent environmental burdens are of major concern.

Producing blue foods through CM systems could circumvent many of the environmental challenges listed above, alleviating pressures on natural ecosystems and giving them time to restore and replenish. Moreover, several blue foods coveted for human diets, including Atlantic bluefin tuna, European eels, and Japanese eels, are classified as threatened or endangered, and their high market value would make it easier for CM products to reach price parity, a known barrier to CM consumer adoption.^34,35^ Together, these make the production of CM blue foods a potentially promising solution to alleviate pressures on wild fish stocks. However, how CM blue foods perform across other environmental lifecycle impact categories remains largely unknown.

First, the small but growing number of LCAs of CM production has focused almost exclusively on livestock and poultry^6,8,10,36–40^ (see section S.1 of the Supporting Information), and their relevance for cultivated blue foods, whose production requires different technologies, inputs, and production protocols, is limited.^41^ For example, blue food cells require lower temperatures compared to mammalian cells, which may affect energy consumption. The colder cultivation environment may also necessitate adjustments in the concentrations of CO_2_ and bicarbonate used to regulate acidity levels, as their solubility increases at lower temperatures (for more, see sections S.1 and S.2 of the Supporting Information).

Second, with few notable exceptions (for example, Kim et al.^8^), past LCAs report results for CM products made solely from cultivated biomass. In practice, however, CM products that blend cultivated biomass with plant-based ingredients (e.g., textured vegetable protein, vegetable oils, spices) are expected to reach technological and economic maturity first, with a few hybrid products already on the market.^42^ As the inclusion of plant-based ingredients could shape energy demand, material inputs, and upstream agricultural burdens, it may affect LCA results of CM products likely to be produced, distributed, and consumed at scale. Thus, biomass-only estimates, while highly informative from a theoretical standpoint, may be less suited for comparisons with conventional animal-based proteins as well as plant-based meat analogs.

Finally, past LCA studies of CM mostly report on global warming (GW) and cumulative energy demand (CED). While the focus on energy and climate impacts makes sense when considering the disproportionate climate impacts of livestock and particularly beef, it may obscure environmental trade-offs. Such trade-offs may be particularly relevant considering the inherent differences between CM and conventional production systems. A more balanced spectrum of environmental impacts is thus needed to compare and understand the potential system-level environmental implications of CM blue food adoption and propose pathways for improvement.

In this study, we use LCA to assess the environmental impacts associated with the production of three hybrid CM blue food products that blend cultivated biomass with other ingredients (primarily plant-based proteins and oils). Examining six impact categories (GW, CED, freshwater and marine eutrophication, freshwater consumption, and land use), we compare the CM products with conventionally produced analogous blue foods and identify environmental hotspots in their production and supply chains. To the best of our knowledge, this study presents the first empirical assessment of the environmental impacts of CM blue food products.

## 2 Methods and Materials

To assess the environmental impacts associated with CM blue food products, compare them against conventional blue foods and explore if and how they can be attenuated, we conducted an attributional LCA following established methodological guidelines and frameworks.^43–47^

LCA models were established for the three hybrid CM products and their conventionally produced analogs: Japanese eel (*Anguilla japonica*), fish sticks, and fish burgers, with the latter two made from sea bream (*Sparus aurata*). CM products were modeled building on primary data from an industry partner, Forsea Foods, Ltd., a leading CM blue foods startup, according to projected specifications. Conventional analogs were modeled based on a combination of primary data from industry experts and existing LCA models as detailed below.

Modeling relied on background data, primarily sourced from three databases: ecoinvent 3.10 (cut-off),^48^ Agri-Footprint 6.3,^49^ and Agribalyse 3.1.1.^50^ An overview of the CM production system is presented below (Figure 1a), and detailed information on data inputs, assumptions, and specific unit processes used in modeling both CM and conventional products are available in the Supporting Information (see sections S.2–S.5 of the Supporting Information). All LCAs reported in this paper were performed using SimaPro (version 9.6.0.1).

**Figure 1:**
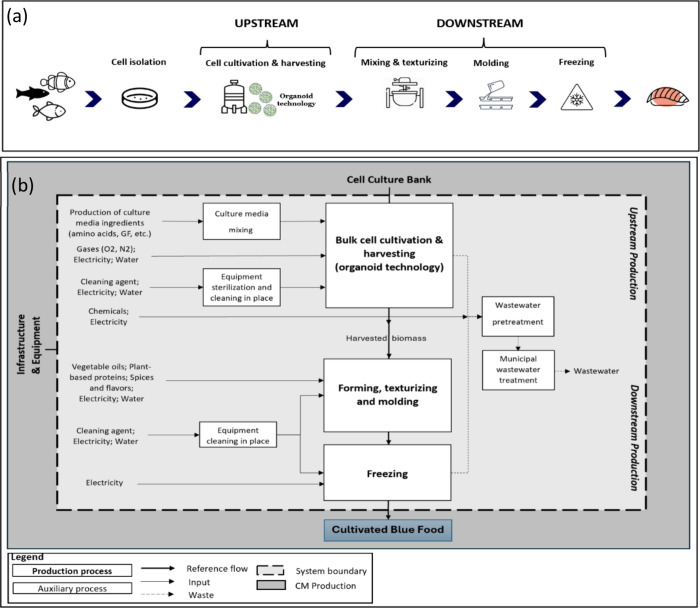
(a) Simplified illustration of CM blue food production process, and (b) system boundaries for CM blue food production. The schematic represents the cradle-to-gate system boundary for CM blue food production, encompassing upstream biomass generation in bioreactors, downstream processing stages, including product formulation and freezing, as well as auxiliary operations such as sterilization and cleaning. The figure also highlights key inputs, including cell culture media components, energy, water, and additional ingredients.

### 2.1 Goal and Scope

The main goal of this work is to assess and compare the environmental impacts associated with the commercial-scale production of CM blue food products to conventional blue foods. In line with previous work,^6,8,10^ we used one kg of final, edible product at the factory gate as the functional unit. The final cultivated products, CM eel (13% protein; 2% fat), CM fish sticks (12% protein; 6% fat), and CM fish burger (11.5% protein; 5.5% fat), are hybrid products made of 30% cultivated organoid biomass (20% protein and 3% fat content) mixed with plant-based oils, textured vegetable proteins (TVP), herbs, spices, and other ingredients, added according to each product’s recipe.

Six environmental impact categories were chosen to facilitate a relevant comparison with past work on CMs and conventional blue foods, including: GW over a 100-year time horizon based on IPCC 2021,^51^ CED based on VDI,^52^ and land use, freshwater consumption, freshwater eutrophication, and marine eutrophication based on ReCiPe 2016 Midpoint­(h).^53^

The system boundaries (Figure 1b) were defined as cradle-to-factory gate for both CM and conventional products. For CM production, the boundaries encompass the full production process, including routine operations such as equipment sterilization and cleaning, due to their material and energy requirements. The isolation of initial cell lines (for CM products) and capital goods (e.g., equipment and facility construction) were excluded from the analysis, following previous LCAs of CMs, which report that their environmental impacts are negligible when considering large-scale production.^10,39^ Packaging was assumed to be similar for both CM and conventional products and was thus also excluded.

### 2.2 CM Blue Food Data

Primary data on the CM production facility, machinery specs, materials, and production protocols were provided by the company in a series of iterative sessions during 2024–2025. These data included projected amounts of material, energy, and waste for all production stages, including biomass production (upstream), and production of the final CM products (downstream). Projected amounts were upscaled from pilot-scale operations based on techno economic analyses and engineering specifications for the company’s planned 15 000 m^2^ production facility in Japan. Some foreground inventory data cannot be disclosed in full for proprietary reasons (e.g., exact media composition, final product recipes; see sections S.2 and S.3 of the Supporting Information for details). All impacts were attributed to the CM products, as this production process did not have any known byproducts with economic value.

#### 2.2.1 Biomass Production

Modeling of biomass production (cell cultivation, upstream process) was based on the company’s patented organoid technology that enables the simultaneous proliferation and differentiation of stem cells into muscle, fat, and connective tissues within self-organized three-dimensional microtissues. For more on the organoid technology [technology readiness level (TRL) 5; biomanufacturing readiness level (BioMRL) 5]; see section S.2 of the Supporting Information.

Biomass production was assumed to include ten SKID systems (self-contained modular systems integrating all required stirred-tank bioreactors and processing equipment), in which cells are grown suspended in a nutrient-rich environment with perfusion over 30-day cycles. Each biomass production cycle begins with bioreactor seeding and includes a perfusion process at 0.5 vessel volumes per day (VVD), where 50% of the bioreactor volume (5 m^3^) is replaced daily with fresh culture media and water. After reaching the target cell density, accumulated cultured cell biomass is harvested daily, while continuous media and water replacement is maintained. Research and Development (R&D) scale trials confirm that this upstream system delivers consistent yields of biomass (15% dry matter content) across various blue food species, supporting its broader applicability for CM blue food biomass production.

All CM products modeled used a similar composition of serum-free culture media, which includes standard optimized R&D scale essential nutrients, including amino acids, glucose, fibroblast growth factors (FGFs), and insulin-like growth factor (IGF-1). The company provided a detailed list of all culture media inputs and their amounts, including 19 different types of amino acids. Six of these amino acids were taken from ecoinvent v3.10 and Agri-Footprint v6.3. For the remaining 13 amino acids for which no production data could be found, we used the average of the above-mentioned as a proxy. FGF and IGF-1 were modeled based on Trinidad et al. (2023)^54^ and all other inputs including glucose, salts, and more, were derived from ecoinvent (see section S.3 of the Supporting Information).

#### 2.2.2 Final Product

After harvesting, the biomass moves on to downstream processing where it is mixed with additional ingredients, texturized, and then frozen to create the final product. Quantities of nonbiomass ingredients, such as TVP, plant-based oils, and spices, were modeled according to each final product’s unique recipe as provided by the company. We used country-specific supply chains for plant-based ingredients when specified and if data were available. For example, US soybeans were used to make TVP, upon which the corresponding product was prioritized in ecoinvent (see section S.3 of the Supporting Information).

#### 2.2.3 Production Equipment Sterilization and Cleaning

For upstream equipment, including bioreactors and media preparation lines, sterilization-in-place was assumed to take place at the end of each 30 day production cycle. This process uses superheated steam produced by a clean steam generator, relying on purified water and energy inputs. Cleaning-in-place for both upstream and downstream equipment, including washing with cleaning agents followed by a water rinse, was also assumed to take place and purified water, cleaning agent, and energy inputs were modeled based on equipment specs and operating hour assumptions.

#### 2.2.4 Water, Energy, and Waste

I. Annual freshwater consumption was calculated based on bioreactor inputs and water required for production equipment cleaning and sterilization. High-purity water is essential throughout the entire CM production process,^15,55–57^ and was modeled by adjusting tap water and electricity of an existing ecoinvent process to Japan.
II. Energy demand for the entire production facility was estimated based on inventories of all machinery, including specs on electricity consumption per unit and the anticipated working hours defined according to production capacity and protocols. When specific information on working hours was unavailable, we conservatively assumed machinery operated continuously year round. The electricity mix modeled in the baseline scenario corresponds to the average electricity supply available at a medium voltage level in Japan.
III. Wastewater volume and its physio-chemical properties were modeled based on primary data provided by our industry partner for two wastewater streams. The first stream is ‘spent media’, meaning water removed from the bioreactors during upstream biomass production. Alongside water, this waste stream also included ammonia, lactate, salts, and residual nutrients whose quantities were calculated based on detailed spent media analysis reports. The second stream is water used for equipment cleaning and sterilization processes. As both streams likely contain relatively high loads of salts and organic content, we assumed that all wastewater required pretreatment before it could be sent to municipal treatment facilities. Pretreatment was modeled as a combined approach involving physicochemical treatment and a membrane bioreactor following Teo et al.^58^ After pretreatment, wastewater was assumed to undergo standard municipal treatment, modeled based on Sinke et al.^10^

#### 2.2.5 CM Data Cross-Validation

Data provided by the company were cross-validated using available literature and additional analysis. For example, in addition to mass balance checks, to confirm media amounts and biomass volume, we also examined spent media analyses reports and calculated the carbon balance (see section S.3 of the Supporting Information). Similarly, to confirm electricity consumption estimates, we examined equipment, operating capacity, and power consumption.

### 2.3 Conventional Blue Food Data

For methodological consistency and inventory data compatibility, we also established LCA models for the analogous conventional blue food products, including wild-caught European eel fillet, fish sticks, and fish burgers made from farmed seabream. European eel was modeled based on primary data collected from fishermen in the Netherlands. Fish sticks and fish burgers were modeled by replacing the fish inputs in existing models with farmed seabream. As a general benchmark, we also added Norwegian farmed salmon fillets using data from Johansen et al.^59^ Economic co-product allocation was used for fillets and co-products (e.g., skins and bones). Detailed information on modeling assumptions and data used for conventional blue foods is available in section S.5 of the Supporting Information.

### 2.4 Sensitivity Analyses

To account for pivotal modeling choices in our LCA, we conducted a series of scenario and sensitivity analyses to explore how variations in key input parameters, modeling assumptions, and methodological choices affect the results. Scenario selection was informed by a combination of factors, including hotspot identification from preliminary results (e.g., amino acids, electricity, TVP, and IGF-1), parameter uncertainty, and practical or operational relevance as reported in previous CM studies (e.g., Kim et al.^8^). Accordingly, some scenarios target assumptions related to unit processes with large contributors (e.g., electricity), some test methodological choices (e.g., coproduct allocation), while others (e.g., production failure) reflect process-level uncertainties relevant to emerging CM systems. The modeling assumptions for the sensitivity analyses are detailed below:

I. Electricity source: Past work as well as preliminary results showed electricity consumption to be a major environmental hotspot in CM production.^6,10,36,39^ Therefore, we explored how reliance on renewable energies instead of the average Japanese grid would affect results. To this end, we constructed a renewable-energy scenario where we assume all electricity demand is met by ground-mounted photovoltaic (solar) systems in Japan.
II. Amino acids in culture media: Previous studies and our preliminary results identified amino acids as a major environmental hotspot in CM production.^16^ However, LCI data for these components are largely unavailable,^13^ and the existing data sets show a wide range of environmental impacts across different amino acids. Therefore, we included a conservative scenario where we used the highest-impact amino acid (l-tryptophan) as a proxy for all amino acids used for production.
III. Production failure: Though biomass production could, in theory, continue indefinitely, to lower the risk of biomass contamination, the company plans to halt upstream production every 30 days for equipment cleaning and sterilization. To explore the effects of a biomass contamination event, in this scenario, we assumed that for the same upstream inputs, production yielded only 11 cycles’ worth of biomass instead of the regular 12. Since downstream inputs are proportionally added to the biomass, such a contamination event would not affect downstream input quantities, as these would be scaled down proportionally.
IV. Plant-based protein source: Preliminary results of the three CM products indicated that due to their share of the final product, downstream ingredients, and particularly TVP, were an environmental hotspot. Although the company indicated that soy-based TVP would likely be sourced from the US, and in light of prior studies showing that pea-based protein can exhibit lower environmental impacts than soy-based protein depending on crop geographic origin and agricultural practices,^61^ we assessed the sensitivity of the results by modeling TVP as being derived from peas grown in Europe and processed in the Netherlands.
V. Co-product allocation: The subdivision of environmental impacts between fillets and co-products (e.g., heads, skin, and bones) in conventional blue food production can meaningfully affect results.^62^ We therefore conducted a sensitivity analysis using mass-based allocation (see section S.5 of the Supporting Information).

While the above-mentioned scenarios cover many critical assumptions (see above and in section S.4 of the Supporting Information), they do not capture the full range of uncertainties associated with CM blue food production. For example, IGF-1 was identified as an environmental hotspot, but could not be critically evaluated due to a lack of data. We subsequently urge additional LCA data collection for this and other supporting processes.

## 3 Results

Our results suggest that environmental impacts of CM blue foods are lower than conventionally produced analogs in terms of GW (2.1–2.3 vs 4.1–9.3 kg of CO_2_ equiv kg^–1^) and CED (39.9–44.7 vs 58.1–127.4 MJ kg^–1^; see Figure 2 and section S.6 of the Supporting Information). With the exception of wild-caught eel, CM blue foods are also associated with lower land use (1.1–1.6 m^2^ a kg^–1^), marine eutrophication (0.9–1.1 g of N equiv kg^–1^) and freshwater eutrophication (0.9–1.3 g of P equiv kg^–1^), compared with conventional blue foods (3.9–18.5 m^2^ a kg^–1^, 186–287 g of N equiv kg^–1^, and 1.6–3.6 g of P equiv kg^–1^, respectively). In terms of water consumption, however, results are mixed. CM eel, CM fish sticks, and CM burgers consume more freshwater (96.7, 152, 142 L kg^–1^, respectively) compared with their analogs (e.g., 44.6 L kg^–1^ for conventional fish burgers), but less than farmed salmon fillets (213 L kg^–1^).

**Figure 2:**
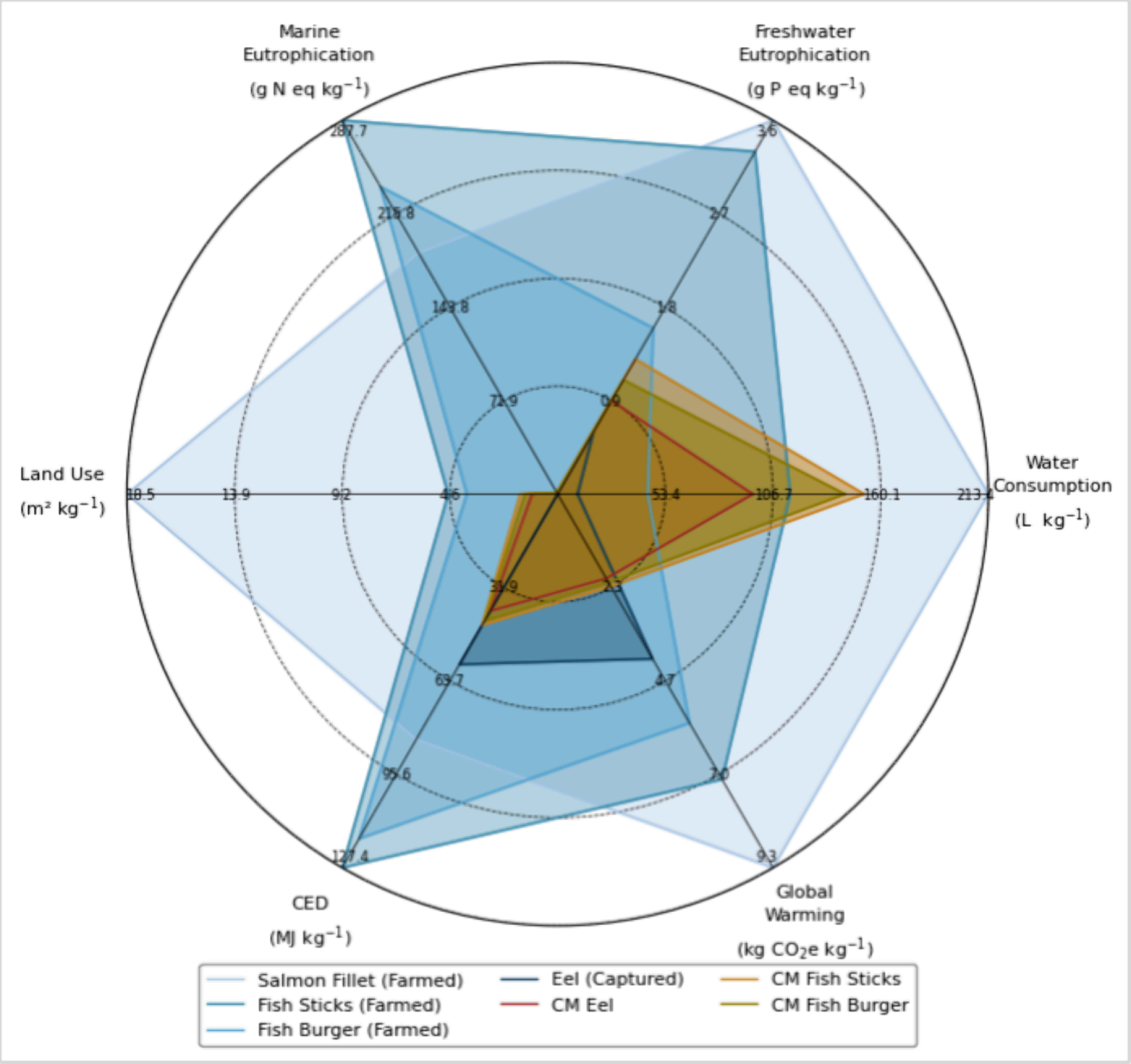
Environmental impacts per kilogram of CM and conventional blue foods. The spider plot shows impacts associated with the production of 1 kg of blue foods in terms of: GW, CED, land use, marine eutrophication, water consumption, and freshwater eutrophication. Seven blue food types are modeled: three CM blue food products (eel, fish sticks, and fish burgers, in red and orange shades) and four conventional counterparts (captured eel, farmed salmon fillet, and fish sticks and burger made with farmed seabream, in blue shades).

When considering our results for CM blue foods against other CM products, the most relevant comparison is with Kim et al.,^8^ who report results for a hybrid-cultivated beef patty. We find that GW, CED, and land use are lower for CM blue compared with the CM beef patty (baseline scenario;^8^ 3.8 kg of CO_2_ equiv kg^–1^, 47 MJ kg^–1^, and 9.5 m^2^ a kg^–1^, respectively; see section S.1 of the Supporting Information), while water consumption is higher for the CM fish sticks and burger, but not for CM eel (110 L kg^–1^). To put our results into context with additional LCAs on cultured meats, we also compare our findings for biomass production (i.e., upstream process only), with those reported by Sinke et al.^10^ who studied several CM products made primarily out of cultivated biomass. Here, too, we find lower impacts for CM blue foods in terms of GW (4.5 kg of CO_2_ equiv kg^–1^), CED (101 MJ kg^–1^), land use (0.4 m^2^ a kg^–1^), and marine eutrophication (1.2 g of N equiv kg^–1^), compared with those reported by Sinke et al. (14.3 kg of CO_2_ equiv kg^–1^, 278 MJ kg^–1^, 2.4 m^2^ a kg^–1^, and 1.3 g of N equiv kg^–1^, respectively, under the baseline-conventional energy scenario^10^). Water consumption (175 L kg^–1^) and freshwater eutrophication (1.5 g of P equiv kg^–1^) are, however, higher for CM blue food biomass (71 L kg^–1^ and 1.4 g of P equiv kg^–1^, respectively). Critically, previously published LCAs may differ from the current analyses in terms of system boundaries, impact assessment methodologies, background data, and other modeling assumptions.

Figure 3, presents GW per kilogram of product for both CM and conventional blue foods, with a breakdown into the relative contributions of feed (i.e., media in CM system), energy, and all other inputs. For CM products, feed (media) accounts for nearly half of GHG emissions, followed by energy use (25% for CM fish burger and CM fish sticks; 27% for CM eel), while the rest are attributed to other inputs. Within the feed category, amino acids account for roughly 34% and IGF-1 for approximately 7% of total GHG emissions, whereasin “other inputs”, TVP is the dominant GHG contributor, responsible for 8–17% of total GHG emissions (for CM eel and CM fish burger, respectively).

**Figure 3:**
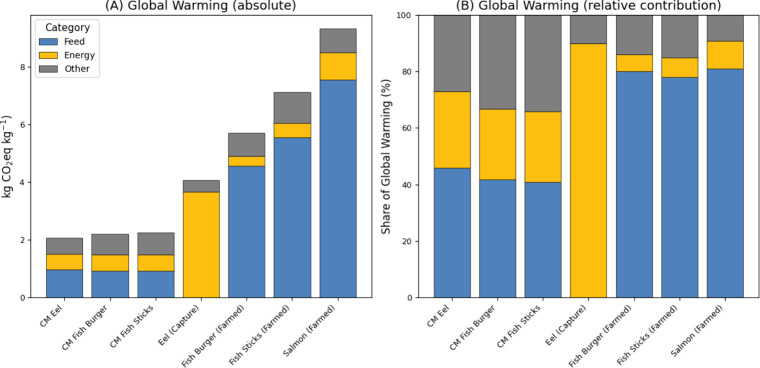
Contribution of feed, energy, and other sources to GW by product. The figure presents contribution to GW impacts (GWP100) for each product in (a) kg of CO_2_ equiv per kg, and (b) relative percentages. Categories are broken down by primary contributing sources: feed (media in the CM system), energy use (during production), and other inputs (plant-based proteins, transport, cleaning agents, fishing nets, etc.).

For conventional products, the contributors to GW impacts differ between captured and farmed systems. For wild-caught eel, emissions are predominantly driven by fuel consumption in fishing vessels, which accounts for approximately 90% of total emissions. For farmed conventional products, such as fish sticks and salmon fillet, feed production is the primary contributor, responsible for 78–81% of total GHG emissions.

### 3.1 Sensitivity Analyses

Figure 4 presents the environmental impacts of various scenarios used to assess variability and uncertainty against the CM eel baseline, alongside results for wild-caught eel as a benchmark. Among all alternatives, the renewable energy scenario delivers the greatest overall benefits, lowering GW to 1.6 kg of CO_2_ equiv kg^–1^ (−24%) and CED to 35.8 MJ kg^–1^ (−10%), with only marginal changes across other categories. In contrast, the intensive amino acid scenario, modeled using the most carbon-intensive amino acid as a proxy for all other amino acid types, results in substantial increases across impact categories: GW rises to 2.9 kg of CO_2_ equiv kg^–1^ (+38%), water consumption to 168 L kg^–1^ (+74%), and CED to 62.6 MJ kg^–1^ (+57%). These findings underscore the growth media’s central role in shaping environmental impacts and highlight the influence of assumptions related to amino acids and growth medium composition.

**Figure 4:**
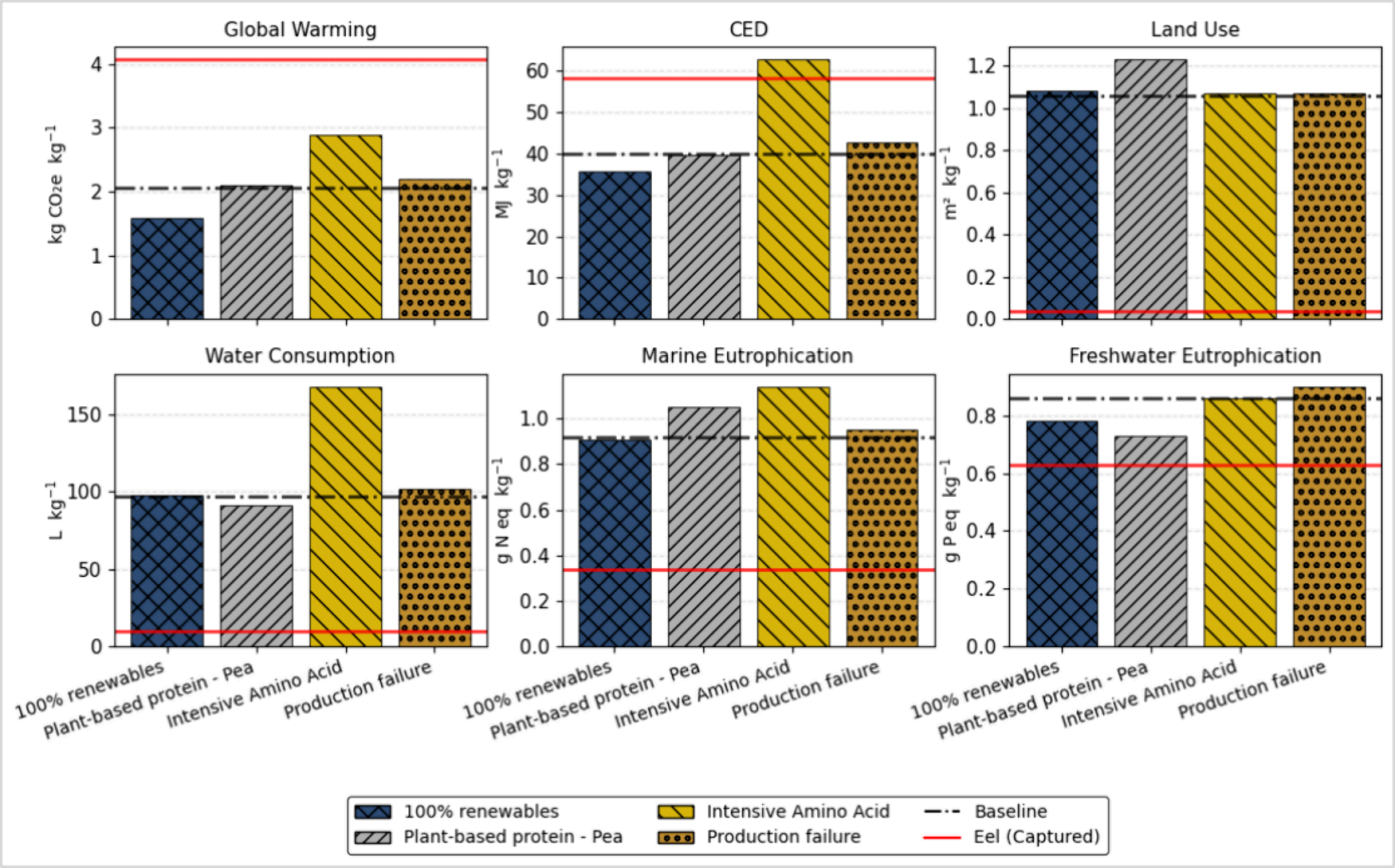
Environmental impacts by production scenario for CM eel. Bars show results for environmental impacts per kg of CM eel under different production scenarios, relative to baseline (CM eel, dashed black line) and conventional wild-caught eel (red line).

The production failure scenario, simulating a lost batch with wasted inputs, increases GW to 2.2 kg of CO_2_ equiv kg^–1^ (+5%) and CED to 42.7 MJ kg^–1^ (+7%), revealing the sensitivity of impacts to inefficiencies in production. Finally, replacing the primary protein source with pea protein produces modest improvements, including reductions in freshwater eutrophication to 0.7 g of P equiv kg^–1^ (−22%) and water consumption to 91 L kg^–1^ (−6%), though increases in land use to 1.2 m^2^ a kg^–1^ (+9%) and marine eutrophication to 1.1 g of N equiv kg^–1^ (+22%). In addition, when mass allocation was applied to the conventional products, CM blue foods were consistently more environmentally benign, with the exception of conventional eel (see section S.6 of the Supporting Information).

## 4 Discussion

Blue foods are an important component of healthy, balanced human diets,^20^ yet their conventional production via aquaculture or wild catch is associated with substantial environmental impacts.^63^ Our findings suggest that producing blue foods through cellular agriculture could offer a more environmental alternative for many commonly consumed aquatic food products, especially for resource-demanding luxury species.^64^ Despite the potential for environmental trade-offs (e.g., higher water consumption compared with captured eel) CM blue foods have lower GHG emissions, require less land, result in less eutrophying emissions, and require less fossil energy compared with conventional production and particularly aquaculture products.

Our results also suggest that CM blue foods may be more environmentally benign compared to other types of cultivated meats.^8,10^ These findings may reflect technological differences between the current production system modeled and past work, as well as lower resource requirements and potentially more resilient cellular structures of aquatic species compared to livestock or poultry. For example, cells of aquatic animals require substantially lower cultivation temperatures than mammalian cells. Moreover, the specific technology examined in the present research relies on a proprietary organoid-based approach that enables simultaneous proliferation and differentiation of stem cells into muscle, fat, and connective tissues within self-organized three-dimensional microtissues. By more closely mimicking natural tissue architecture, this technology potentially reduces culture media requirements and external growth factor inputs, and eliminates the need for scaffolding materials used to create three-dimensional structures.^10,65^ However, these results may also stem from differences in impact assessment methods employed, as well as the underlying meaning of the functional unit “1 kg”. Unlike most past CM LCAs which examined products made mostly out of cultivated biomass, the final CM products examined here are hybrid ones and include large shares of noncultured ingredients (e.g., plant proteins and oils). While the comparison with the conventional analogs is appropriate (after all, fish sticks and burger also contain plant-based ingredients), our results for biomass cultivation may be more suitable for direct comparisons with studies that examine CM products made predominantly from cultivated biomass.

Nonetheless, despite differences in production technologies, protocols, and even species, in line with previous work, we find that the major environmental hotspots include media production, energy use, and water consumption. These findings highlight key priorities for improving the environmental performance of CM blue foods, including the development of lower-impact media formulations (e.g., alternative amino acids), sourcing TVP from carbon-efficient production systems, relying on renewable electricity sources, and optimizing process water use.

Integrating recycling technologies to recover and reuse water and valuable nutrients or compounds from spent media could reduce reliance on virgin inputs, lower waste generation, and decrease overall resource intensity, thereby improving environmental performance. At the same time, it could also lower production costs and allow CM products to compete more effectively with conventional animal-based foods. Advancing research and technological innovation in this area will therefore be critical for the long-term sustainability of CM production.

Finding appropriate waste treatment strategies that comply with environmental and public health regulations is also important, given the potential influence of waste management on the overall performance of CM production.^15,39^ While most previous studies did not explicitly address CM wastewater treatment, our analysis suggests that these should not be overlooked. Specifically, results from spent media analyses indicate that the physicochemical properties of wastewater coming from CM production would likely require pretreatment of all effluents before they can be sent to municipal treatment facilities. Future research should therefore systematically characterize the major waste streams generated in CM production and evaluate appropriate treatment pathways, while also assessing whether recycling could cost-effectively reduce both material intensity and wastewater outputs.

Our findings are subject to several limitations. Importantly, most of the data were provided by our industry partner based on projected specifications for their full-scale facility rather than one already in operation, which introduces some uncertainty into our results. While the iterative nature of the data compilation, the cross-validation performed using both external and internal data sources, and the production failure scenario lend strength to our results, our models are still based on technology that is not yet fully mature. As the industry develops, production efficiency may change; therefore, research utilizing actual production data, ideally covering several years, would be needed to confirm the main findings.^44^ Future research could also examine the impacts related to infrastructure, cell sourcing, and large-scale media production,^40,66^ and use market data to inform modeling choices.

Our choices regarding system boundaries, data, and underlying assumptions may not fully capture the complexity of real-world production. For example, most amino acids were modeled based on an average, as granular data for each specific amino acid type are currently unavailable. Moreover, many of the key ingredients of the culture media come from supply chains that currently cater mostly to pharmaceutical-grade products and animal supplements. As demand from the CM industry expands, it is likely that new CM-specialized supply chains will emerge, and their environmental impacts may differ from those currently supplying the CM industry.

Most importantly, perhaps, at the system level, CM systems may be uniquely positioned to ensure continued supply of otherwise depleted blue foods while alleviating pressures on natural habitats and allowing stocks to replenish. Although we did not assess such impacts directly, we hypothesize that the greatest environmental potential of CM systems may be in reducing demand for species that are listed as endangered, rather than for those that are found in abundance (e.g., livestock or poultry). Given that farming of many aquatic species is currently impossible, they are overexploited and become endangered, limiting their supply and raising their market price high enough that CM products become cost competitive. While more work directly examining how such dynamics could affect the overexploitation of conventional blue foods is needed, prioritizing the production of high-impact, premium CM blue foods, such as eels, blue-finned tuna, or lobsters, could hold strategic advantages that maximize sustainability gains.

In light of inherent differences between CM and conventional blue food production systems, CM products could also help to address unique food safety concerns, such as the rising presence of contaminants in conventional blue foods including heavy metals, forever chemicals, microplastics, and antibiotics, all of which are harmful to human health.^67^ More work is therefore needed to shed light on the nutritional aspects of CM blue foods and to examine how they compare with conventional ones. In addition, while not part of this study, factors such as consumer acceptance, palatability, health, regulation, and of course, costs will likely dictate whether future CM products will replace conventional blue foods, or if they will be consumed in addition to conventional ones. Since the environmental benefits of CM blue foods will be achieved only if they effectively replace blue foods and lower demand for their production, it is essential to understand and shape the system-level conditions under which substitution, rather than supplementation, is likely to occur.

Ultimately, the transition toward CM blue foods could mark a pivotal step in transforming blue food production from extractive to regenerative, aligning human nutrition with planetary boundaries. Critically, our results suggest that such a transition may involve trade-offs, underscoring the importance of evaluating novel foods across multiple impact categories rather than relying on GW as a proxy for environmental performance.

## Supplementary Material





## Notes

Additional methodological details and explanations of assumptions (PDF)
Detailed LCI data and input parameters used in the analysis (XLSX)
