# Supplementary material for: Environmental Impacts of Cultivated (Lab-Based) Blue Foods
Source: Environ Sci Technol. 2026 May 26;60(22):15771–80. doi: 10.1021/acs.est.6c02274 (PMC13262027; doi:10.1021/acs.est.6c02274)
Supplement: Supplementary file 1 [file es6c02274_si_001.pdf]

Supporting Information

Environmental impacts of cultivated (lab-based) blue foods

Shira Shabtai[1], Tamar Makov*[1,2], Alon Shepon[3,4], & Patrik JG Henriksson[5,6]

[1]Management Department, Ben-Gurion University of the Negev
[2]School of sustainability and climate change, Ben-Gurion University of the Negev
[3] The Steinhardt Museum of Natural History, Tel-Aviv University
[4]The Department of Public Policy, the Faculty of Social Sciences, Tel Aviv University
[5]Institute of Environmental Sciences (CML), Leiden University, the Netherlands
[6]Stockholm Resilience Centre, Stockholm University, Stockholm, Sweden

S.1. Published findings from previous CM LCA studies

Table 1 presents a summary of published findings from previous cultivated meat LCA studies alongside the results of the present study.

All previous CM LCA studies, alongside this study, followed attributional LCI principle and cradle-to-gate system boundaries.

Table 1: Summary of published findings from previous cultivated meat LCA studies alongside the results of the present study. Updated from Blackstone et al., 2025[1].

Study	Functional Unit (FU)	Protein Content	Global Warming (CO_2eq kg-1)	CED (MJ kg-1)	Land use (m2a eq kg-1)	Water (L kg-1)	Impact Method	Locations	Details
Tuomisto and Teixeira de Mattos (2011)[2]	1 kg of cultivated biomass (muscle cells); 30% dry matter	19% protein	1.9-2.2	26-33	0.19-0.23	367-521	IPCC 2006, Primary energy, Land occupation, Water Footprint Network	Spain, US, Thailand	Stem cells from unspecific animal embryo; 60-day production cycle

Smetana et al. (2015)[3]	1 kg finished product (ready to eat)	-	23.9-24.6	291-373	0.4-0.8	NA	ReCiPe Endpoint (H) V1.08, IMPACT 2002+	Global	Modeled based on data from Tuomisto and Teixeira de Mattos (2011)[2]
Mattick et al. (2015)[4]	1 kg cultivated biomass (CHO cells); 17% dry matter	7% protein	3-25	44-316	2.9-8.5	NA	CED, eco footprint, CML 01	US	Chinese hamster ovary (CHO) cells; Eight-day production cycle (5 proliferation, 3 differentiation)
Tuomisto et al. (2022)[5]	1 kg cultivated biomass (C2C12 cells); 30% dry matter	20% protein	4.9-25.2	94-533	1.8-6.9	120-540	ReCiPe 2016 Midpoint, CED	UK	Immortalized mice cells (C2C12); 23-day production cycle (16 proliferation, 7 differentiation)
Kim et al. (2022)[6]	1 kg hybrid cultivated beef burger (novel burger), 17% cultivated biomass in final product	-	1.93-4.09	47	9.5	110	ReCiPe 2016 Midpoint (E), CML-IA, CED,	US	Bovine cells - muscle and fat
Sinke et al. (2023)[7]	1.1 kg, including 1 kg cultured biomass and 0.1 kg of edible scaffold; 20-30% dry matter	18-25% protein	2.2-24.8	164–278	2.41–2.48	71.2-85.7	ReCiPe 2016 Midpoint, CED	Global	Terrestrial farmed animals cells ("meat cells"); 42-day production cycle
Risner et al. (2025)[8]	1 kg cultivated biomass (CHO cells); 30% dry matter	30% dry matter content (70% protein, 15% lipids, 10% carbohydrates, 5% nucleic acids)	12.3-1508	NA	NA	NA	TRACI 2.1	Global	Chinese hamster ovary (CHO) cells
**This Paper**	1 kg CM blue food, 30% cultivated	11.5-13% protein	2.1-2.3	39.9-44.7	1.1-1.6	96.7-151.9	IPCC 2021 (GWP100);	JP	Stem cells from eel embryo and seabream

	biomass in final product; 15% dry matter						ReCiPe 2016 Midpoint (H)		embryo; 30-day production cycle
	1 kg of cultivated biomass	20% protein	4.5	100.7	0.4	175.1			

S.2. Cultivated blue food technology

The production of CM blue foods leverages a proprietary organoid-based approach, adapted from biomedical tissue engineering applications[9], which represents an advancement over traditional cultured meat systems[10]. Unlike conventional CM production methods, which typically involve two distinct stages, beginning with the expansion of proliferating cell lines as single cells using scaffolds and exogenous growth factors, and subsequently their differentiation into muscle or fat cells - the proprietary organoid-based technology developed by Forsea enables the simultaneous proliferation and differentiation of stem cells into muscle, fat, and connective tissues within self-organized three-dimensional microtissues. This design mirrors natural tissue architecture more closely and enables endogenous growth factor production, thereby reducing dependency on externally supplied additives and eliminating scaffold materials. By integrating embryonic stem cells in a single culture, the organoid approach offers potential reductions in media consumption, material inputs, and overall production costs, while also enhancing structural and nutritional complexity of the biomass. At the intermediate development stage corresponding to Technology Readiness Level (TRL 5-7) and Biomanufacturing Readiness Level (BioMRL 5-7), this technology has been successfully demonstrated under controlled laboratory conditions[11,12].

S.3. CM blue foods data

Detailed unit processes for the three CM products, including data and assumptions related to water consumption, ultrapure systems, sterilization-in-place (SIP), and cleaning-in-place (CIP), are provided in the Supporting Information (SI) tables (T.7-T.13), along with general system characteristics (T.14), electricity consumption data (T.15), and additional assumptions related to spent media analysis, annual carbon balance of cultivated biomass production, and wastewater pre-treatment modeling (T.16-T.19). Certain input data are confidential and therefore not publicly disclosed.

S.3.1. Spent media analysis and carbon balance

Spent media analysis, along with the assumptions applied in the carbon balance estimations for annual cultivated biomass production (i.e., upstream bulk production), is presented in SI tables (T.17-T.18)[13–19]. The carbon conversion efficiency (output/input) was estimated at 47%.

S.3.2. Wastewater pretreatment data

A pre-treatment system adapted from Teo et al. (2023)[20] was modeled using a combined physicochemical and membrane bioreactor (see T.19). The system modeled accounts for electricity and main chemical inputs, and final discharge was modeled using an ecoinvent proxy for food-industry wastewater (Wastewater from potato starch production, RoW). Note,

no recycling or water recovery processes were assumed in the CM blue food production system.

S.3.3. Data quality

The LCA inventory data covers all relevant processes and technologies across the cradle-to-gate supply chain. Data quality for CM product unit processes was evaluated based on the updated pedigree matrix[21], with rankings reflecting the extent to which data rely on assumptions, proxies, or methodological constraints that may affect the robustness of the results (see SI tables - T.6). Foreground unit processes were generally rated higher because they are derived from company-specific measurements or process-based modeling. According to LCA data quality guidance[21], such primary data are considered more reliable due to transparency in data generation and internal validation (e.g., operational protocols, mass balance). In contrast, background datasets rely largely on databases such as ecoinvent, where methodological consistency is high but representativeness may be limited by generic assumptions, geographic averaging, or older data.

For the upstream culture media, reliability is medium-high due to reliance on secondary databases (ecoinvent and Agri-Footprint); completeness is medium, with coverage of essential nutrients but gaps for 13 amino acids filled by averaging; temporal quality is high as datasets reflect recent measurements based directly on company data from 2025; geographical representativeness is medium-high, as main datasets come from multiple global sources; technological representativeness is medium-high (industrial scale uncertain), and precision is medium given reliance on average values for missing amino acids (sensitivity analyses were applied).

For culture media mixing, reliability and completeness are medium-high (inventories rely on primary data from the company regarding volumes and water use); temporal representativeness is high (company data from 2025); geographical representativeness is medium-high (modeled with Japanese energy mix but global water data); technological representativeness is medium-high (industrial scale uncertain); and precision is medium-high, with sensitivity analyses applied.

For bulk cultivation production (upstream), reliability is medium-high based on primary process data (VVD rates, harvest rates, SIP/CIP schedules); completeness is medium-high as operational and cleaning are covered; temporal representativeness is high (2025 protocols); geographical representativeness is medium-high (modeled with Japanese energy mix but global water data); technological representativeness is medium-high (pilot scale evaluations for commercial scale); precision is medium (bioprocess variability- sensitivity analyses were applied).

For downstream CM products (eel, fish sticks, fish burger), reliability is medium-high, relying on recipes combining primary data (biomass shares, texturization, TVP composition) with secondary data; completeness is medium-high, as energy, mixing, and ingredient flows are represented. but flavorings are simplified; temporal representativeness is high (2025 protocols); geographical representativeness is medium-high (main ingredients are EU/US-based datasets); technological representativeness is medium-high (industrial scale uncertain); and precision is medium, sensitivity analyses were applied.

S.4. Sensitivity Analysis

The following section outlines the sensitivity analysis scenarios examined in this study. Detailed changes in unit processes are presented in SI tables (T.13).

S.4.1. 100% renewables

Electricity demand is modeled as supplied by ground-mounted photovoltaic (solar) systems in Japan using the corresponding ecoinvent process. This substitution covers electricity requirements for all production stages, including the ultrapure water system and wastewater pre-treatment process.

S.4.2. Intensive Amino Acid Scenario

The most impact-intensive dataset, L-tryptophan, was used as a proxy for the 13 amino acids lacking unit process data. Environmental impacts of the six amino acids, obtained from Agri-Footprint and ecoinvent, are provided in the SI tables (T.16).

S.4.3. Production Failure Scenario

An extreme failure scenario was considered, where it is assumed that inputs intended for 12 production cycles yield biomass equivalent to only 11 cycles, meaning that the same upstream inputs would produce 92% of the baseline biomass. Because downstream input quantities are scaled to the amount of biomass produced, such a failure would not result in wasted downstream ingredients, as these are adjusted proportionally to the reduced upstream biomass output.

S.4.4. Planet-based protein – pea

TVP was modeled as derived from peas cultivated in the Netherlands, represented by the corresponding Agri-Footprint process. Transportation was adjusted accordingly, including an update of the ‘freight, sea’ distance from Rotterdam to Tokyo.

S.4.5. Coproduct allocation

See Section S.5 for further details on the sensitivity analysis applying mass-based allocation to conventional products.

S.5. Conventional Blue Foods Data

The functional unit was defined as 1 kg of either fillet cut or final processed product. Conventional blue food systems were modeled using cradle-to-gate system boundaries, excluding capital goods (e.g., equipment and facilities), packaging and distribution beyond the factory gate. Detailed data, unit processes, and data quality scoring are provided in the SI tables.

S.5.1 Wild-caught EU eel (NL)

LCI for wild-caught eel in the Netherlands is based on primary data collected through interviews with fishermen in Urk (conducted on 21 March 2025). On average, one fishing vessel captures approximately 1,200 kg of eel per week using 500 liters of diesel and 30 nets, with each net yielding 20 kg of eel over a three-day period. The fleet consists of five vessels operating with a total of 150 nets. The fishing season extends from May to September, and eel sizes range from 0.25 kg to 1.5 kg. The inventory for fishing net materials and energy inputs was based on Karadurmuş and Bilgili (2024)[22]. Electricity use for processing at slaughter plants is based on Eliassen, De Rosa, & Schmidt (2021)[23] and electricity use for freezing is based on Johansen et al. (2022)[24]. An edible yield of 40% was estimated based on a combination of fisher-reported values and Agribalyse datasets for Conger, fillet, raw and Conger, residues, raw (France). Detailed input data are provided in the supporting tables (T.20-T.22). No primary data was available for by-product allocation. Therefore, the baseline model applied an economic allocation factor of 98.2%, based on available data for Salmon (see S.5.4). In addition, a sensitivity analysis using mass-based allocation was performed, allocating 40% of impacts to the edible portion.

S.5.2. Fish sticks (from farmed sea bream)

To enable a consistent comparison between conventional and cultivated fish sticks, the ecoinvent process "frozen fish sticks production, hake" was adapted by substituting the original input ("Fish block, hake {GLO} | market for fish block, hake | Cut-off, U") with "Black seabream, fillet, raw, at processing {FR} U". This substitution reflects the use of seabream rather than frozen hake blocks as in the original process.

To align with the focus on aquaculture-sourced seabream, the original seabream fillet input (based on captured fish) was further modified. The updated inventory replaces it with "Sea bass or sea bream, 200–500g, conventional, in cage, at farm gate {FR} U", representing farmed production. This aquaculture process was adjusted to match the system boundaries of the current study, excluding equipment-related inputs. Based on the Agribalyse dataset for black seabream fillet, the edible yield was estimated at 42%. As this dataset does not include an allocation procedure, the baseline model applied an economic allocation factor of 98.2%,

based on data for salmon (see S.5.4). A sensitivity analysis using mass-based allocation was also conducted, assigning 42% of impacts to the edible portion (see SI Table T.23).

S.5.3. Fish Burger (from farmed sea bream)

To facilitate a consistent comparison with cultivated seabream-based fish burgers, the Agribalyse process "Fish burger, fast foods restaurant, at plant {FR}" was modified by replacing the original input of "Cod, fillet, raw, at processing {FR} U" with "Black seabream, fillet, raw, at processing {FR} U". This adapted process reflects the use of seabream fillet as the primary ingredient. The adjusted process also incorporates energy use for freezing, informed by data from SINTEF's analysis of Norwegian salmon processing[24].

To better represent aquaculture-based production, the "Black seabream, fillet, raw, at processing {FR} U" process (based on wild-caught fish) was further adjusted. It was substituted with "Sea bass or sea bream, 200–500g, conventional, in cage, at farm gate {FR} U", representing farmed seabream. This aquaculture dataset was modified to align with the study's system boundaries, excluding equipment-related inputs. Based on the Agribalyse dataset for black seabream fillet, the edible yield was estimated at 42%. Consistent with the fish sticks modeling approach, the baseline model applied an economic allocation factor of 98.2%. Sensitivity analyses were subsequently conducted using a mass-based allocation, assigning 42% of impacts to the edible portion (see SI Table T.24).

S.5.4. Norwegian salmon (farmed)

Norwegian farmed salmon produced in a Recirculating Aquaculture System (RAS) was modeled based on Johansen et al. (2022)[24], with feed and aquaculture operations assessed separately (see SI tables T.25-T.27). The edible portion was estimated using Agribalyse datasets: "Farmed salmon, conventional, head on, gutted {NO} U" and "Salmon, fillet, raw, consumption mix {FR} U". Conversion mass ratios were applied according to PEFCR Marine Fish guidelines[25] - from round salmon to Head-On Gutted (HOG) salmon (0.83), and from HOG to B-trim fillet (0.65), yielding an overall fillet yield of 54%. By-product treatment and allocation were based on the Agribalyse "Salmon, fillet, raw, consumption mix {FR} U" process, which assumes economic allocation of 98.2% to the fillet and 1.8% to co-products. Notably, 98% of by-products from salmon slaughter and processing in Norway are utilized in various ways[24].

S.5.5 Data Quality

Data quality was assessed using the pedigree matrix[21] (SI Table T.6). For wild-caught eel, primary activity data (diesel, nets, catches) are highly reliable, whereas background processes (e.g., net production) are medium. Completeness is medium-high, covering energy and material inputs but with some uncertainty in by-product allocation. Temporal and

geographical representativeness are medium-high, reflecting 2025 interviews and Dutch fishing conditions, while technological representativeness is medium, excluding detailed vessel construction. Precision is medium due to fisher-reported variability.

For farmed seabream products (fish sticks, fish burgers), data reliability is medium-high, based on ecoinvent and Agribalyse datasets. Completeness is medium, covering edible yield, with by-product allocation requiring sensitivity analysis. Temporal representativeness is medium-high, while geographical and technological representativeness are medium, reflecting EU-based datasets and adjustments for aquaculture boundaries. Precision is medium, reflecting reliance on secondary data.

For Norwegian farmed salmon, production data from SINTEF and Agribalyse are medium-high in reliability. Completeness, temporal, and geographical representativeness are medium-high, covering feed, aquaculture, processing, and by-products. Technological representativeness is medium-high, reflecting detailed RAS systems and PEFCR conversions. Precision is medium-high, with some default allocations from Agribalyse and PEFCR.

S.6. Complete impact assessment results for cultivated and conventional blue foods

Comprehensive impact assessment results for the CM baseline products, scenarios, and conventional blue foods are provided in the SI tables (T.1) per 1 kg of final product, covering indicators from IPCC (2021), ReCiPe 2016 M(h), and CED. For conventional products, additional sensitivity analyses were performed using a mass allocation approach (T.2). GW breakdown into relative contributions per kg of product for both CM and conventional blue foods is presented in T.3-T.4. Additionally, the full set of ReCiPe 2016 M(h) indicators for CM and conventional products is presented in T.5.

SI – references

(1) Blackstone, N. T.; Pavlova, A.; Trinidad, K. R.; Nikkhah, A.; Sinke, P.; Heller, M.; Duncan-Duggal, J.; Ridoutt, B.; Smetana, S.; Makov, T.; Shabtai, S.; Green, A.; Barnes, W.; Bhattarai, I.; Goyal, S.; Imholz, N.; Meshulam, T.; Nadar, C. G.; Norris, G. A.; Quandt, J.; Ronco, N.; Tuomisto, H. L. Guidelines for Environmental Life Cycle Assessment of Cultivated Meat. *International Journal of Life Cycle Assessment* **2025**. https://doi.org/10.1007/s11367-025-02562-4.

(2) Tuomisto, H. L.; Teixeira de Mattos, M. J. Environmental Impacts of Cultured Meat Production. *Environ. Sci. Technol.* **2011**, *45* (14), 6117–6123. https://doi.org/10.1021/es200130u.

(3) Smetana, S.; Mathys, A.; Knoch, A.; Heinz, V. Meat Alternatives: Life Cycle Assessment of Most Known Meat Substitutes. *Int. J. Life Cycle Assess.* **2015**, *20* (9), 1254–1267. https://doi.org/10.1007/s11367-015-0931-6.

(4) Mattick, C. S.; Landis, A. E.; Allenby, B. R.; Genovese, N. J. Anticipatory Life Cycle Analysis of In Vitro Biomass Cultivation for Cultured Meat Production in the United States. *Environ. Sci. Technol.* **2015**, *49* (19), 11941–11949. https://doi.org/10.1021/acs.est.5b01614.

(5) Tuomisto, H. L.; Allan, S. J.; Ellis, M. J. Prospective Life Cycle Assessment of a Bioprocess Design for Cultured Meat Production in Hollow Fiber Bioreactors. *Sci. Total Environ.* **2022**, *851* (Pt 1), 158051. https://doi.org/10.1016/j.scitotenv.2022.158051.

(6) Kim, S.; Beier, A.; Schreyer, H. B.; Bakshi, B. R. Environmental Life Cycle Assessment of a Novel Cultivated Meat Burger Patty in the United States. *Sustainability*. 2022. https://doi.org/10.3390/su142316133.

(7) Sinke, P.; Swartz, E.; Sanctorum, H.; van der Giesen, C.; Odegard, I. Ex-Ante Life Cycle Assessment of Commercial-Scale Cultivated Meat Production in 2030. *Int. J. Life Cycle Assess.* **2023**, *28* (3), 234–254. https://doi.org/10.1007/s11367-022-02128-8.

(8) Risner, D.; Negulescu, P.; Kim, Y.; Nguyen, C.; Siegel, J. B.; Spang, E. S. Environmental Impacts of Cultured Meat: A Cradle-to-Gate Life Cycle Assessment. *ACS Food Science & Technology* **2025**, *5* (1), 61–74. https://doi.org/10.1021/acsfoodscitech.4c00281.

(9) Yang, S.; Hu, H.; Kung, H.; Zou, R.; Dai, Y.; Hu, Y.; Wang, T.; Lv, T.; Yu, J.; Li, F. Organoids: The Current Status and Biomedical Applications. *MedComm (2020)* **2023**, *4* (3), e274. https://doi.org/10.1002/mco2.274.

(10) Kim, S.; Jeong, Y.; Jo, H.; Park, Y. G.; Moon, S. H. Cultured Meat: Advances in Stem Cell Biology, Tissue Engineering, and Bioprocess Optimisation for Scalable and Sustainable Production—a Review. *International Journal of Food Science and Technology*. Oxford University Press July 1, 2025. https://doi.org/10.1093/ijfood/vvaf220.

(11) Smanski, M. J.; Aristidou, A.; Carruth, R.; Erickson, J.; Gordon, M.; Kedia, S. B.; Lee, K. H.; Prather, D.; Schiel, J. E.; Schultheisz, H.; Treynor, T. P.; Evans, S. L.; Friedman, D. C.; Tomczak, M. Bioindustrial Manufacturing Readiness Levels (BioMRLs) as a Shared Framework for Measuring and Communicating the Maturity of Bioproduct Manufacturing Processes. *J. Ind. Microbiol. Biotechnol.* **2022**, *49* (5), kuac022. https://doi.org/10.1093/jimb/kuac022.

(12) Kimmel, W. *Technology Readiness Assessment Guide: Best Practices Guide*; 2020. https://ntrs.nasa.gov/api/citations/20205003605/downloads/%20SP-20205003605%20TRA%20BP%20Guide%20FINAL.pdf.

(13) Alberts, B.; Johnson, A.; Lewis, J.; Raff, M.; Roberts, K.; Walter, P. *Molecular Biology of the Cell*, 4th ed.; Garland Science, 2002.

(14) International Union of Pure; (IUPAC), A. C. Standard Atomic Weights. 2024. https://iupac.org.

(15) Lehninger, A. L.; Cox, M. M. *Lehninger Principles of Biochemistry*, 7th ed.; W.H. Freeman, 2017.
(16) Nelson, D. L.; Cox, M. M. *Lehninger Principles of Biochemistry*, 7th ed.; W.H. Freeman, 2017.
(17) National Center for Biotechnology Information (NCBI). Biochemistry (5th Ed., Berg, Tymoczko, & Stryer). 2004. https://www.ncbi.nlm.nih.gov/books/NBK9879/.
(18) National Center for Biotechnology Information (NCBI). PubChem Database. 2024. https://pubchem.ncbi.nlm.nih.gov.
(19) National Center for Biotechnology Information (NCBI). Lactic Acid. PubChem. 2024. https://pubchem.ncbi.nlm.nih.gov/compound/Lactic-acid.
(20) Teo, C. J.; Karkou, E.; Vlad, O.; Vyrkou, A.; Savvakis, N.; Arampatzis, G.; Angelis-Dimakis, A. Life Cycle Environmental Impact Assessment of Slaughterhouse Wastewater Treatment. *Chemical Engineering Research and Design* **2023**, *200*, 550–565. https://doi.org/10.1016/j.cherd.2023.11.016.
(21) *Guidance on Data Quality Assessment for Life Cycle Inventory Data*. www.epa.gov/research.
(22) Karadurmuş, U. Environmental Impacts of Synthetic Fishing Nets from Manufacturing to End-of-Life. *Mar. Pollut. Bull.* **2024**, *185*, 114278. https://doi.org/10.1016/j.marpolbul.2023.114278.
(23) Eliassen, J. L.; De Rosa, M.; Schmidt, J. *Life Cycle Assessment (LCA) of Kangamiut Seafood Products*; 2021. https://lca-net.com/files/Report_Kangamiut_20211022_2.pdf.
(24) Johansen, U.; Nistad, A. A.; Ziegler, F.; Mehta, S.; Langeland, M.; Wocken, Y.; Hognes, E. S. *Greenhouse Gas Emissions of Norwegian Salmon Products*; 2022. https://hdl.handle.net/11250/3044084.
(25) European Commission DG SANTE. *Product Environmental Footprint Category Rules (PEFCR) for Unprocessed Marine Fish Products*; Brussels, Belgium, 2021. https://ec.europa.eu/environment/eussd/smgp/PEFCR_OEFSR_en.htm.
